# Experiences of LGBTQIA+ medical students: gaps and challenges during preclinical education

**DOI:** 10.1186/s12909-026-08966-6

**Published:** 2026-03-10

**Authors:** Samantha Temucin, Natalie Florescu, Paige Varin, LeeAnn Tanaka, Robert Bettiker, Erin N. Harrop, Kendrin Sonneville

**Affiliations:** 1 Philadelphia College of Osteopathic Medicine, Philadelphia, PA USA; 2 University of Vermont Medical Center, Burlington, VT USA; 3 Department of Family Medicine, Philadelphia College of Osteopathic Medicine, Philadelphia, PA USA; 4 Section of Infectious Diseases, Temple University, Philadelphia, PA USA; 5 Graduate School of Social Work, University of Denver, Denver, CO USA; 6 Department of Nutritional Sciences, University of Michigan School of Public Health, Ann Arbor, MI, USA

**Keywords:** LGBTQIA+, Medical students, Medical education, Campus climate, Sexual and gender minority

## Abstract

**Background:**

While attempts to diversify the physician workforce are ongoing, limited research exists exploring the experiences LGBTQIA+ medical students during their preclinical training. The purpose of this study is to examine how interpersonal interactions, campus climate, and the availability of campus support influence the well-being of LGBTQIA+ medical students across U.S. medical schools during their preclinical education.

**Method:**

Medical students self-identifying as LGBTQIA + and enrolled in either Doctor of Medicine (MD) or Doctor of Osteopathic Medicine (DO) institutions in the U.S. in 2024 completed an online, 136-item survey. Participants were asked about preclinical medical school experiences, perceptions of their campus climate and curriculum, and perceived level of impact experiences had on their well-being.

**Results:**

Four hundred fourteen completed records were analyzed, representing LGBTQIA+ medical students across MD (*n* = 295, 71.3%) and DO (*n* = 119, 28.7%) programs, including 309 cisgender (74.6%) and 97 (23.4%) transgender or gender-expansive (TGE) students. The majority of participants indicated that their school fell short in providing supportive diversity initiatives, LGBTQIA+ specific spaces, positive LGBTQIA+ representation, and adequate responses to discrimination. Most participants also reported that professors did poorly at using inclusive terminology and advocating for LGBTQIA+ inclusion. TGE students consistently rated their school, classmates, professors, and campus climate lower than cisgender students, and were significantly more likely to report differential treatment (55.7% vs. 23.3%, *p* < .001) and overt harassment (23.7% vs. 4.5%, *p* < .001). Students in red states or rural settings reported significantly less inclusivity and campus support than those in blue states or urban settings.

**Conclusions:**

LGBTQIA+ medical students face significant barriers to well-being, including adverse campus experiences, inadequate support, and limited inclusivity. This study highlights an acute need for interventions that improve the preclinical environment for LGBTQIA+, and particularly TGE, medical students.

**Supplementary Information:**

The online version contains supplementary material available at 10.1186/s12909-026-08966-6.

## Background

Lesbian, gay, bisexual, transgender, queer, intersex, and asexual (LGBTQIA+) patients face numerous health disparities and barriers to care, yet LGBTQIA+ identities remain significantly underrepresented among physicians [1]. As medical schools attempt to address this gap, it is crucial to identify the unique challenges, barriers, and supports that influence these students’ success and future careers. Understanding these factors is not only an issue of equity but also one of educational and workforce development; an inclusive and supportive learning environment directly affects student well-being, academic achievement, and retention, ultimately shaping the diversity and competence of the physician workforce.

Numerous studies have highlighted that LGBTQIA+ medical students, residents, and physicians frequently face discrimination, microaggressions, and bullying [2–12]. Beyond direct harm, exposure to marginalizing language or witnessing peers and professors discuss LGBTQIA+ identities in stigmatizing ways can erode students’ sense of belonging and trust in mentors [2, 3]. Furthermore, LGBTQIA+ topics remain underrepresented in medical school curricula, compounding these challenges during didactic years [13–19]. The limited and inconsistent inclusion of LGBTQIA+ health content, particularly regarding the care of transgender and gender-diverse patients, creates a gap between curricular intent and practice [13–17]. When students perceive a lack of institutional support or inclusive education, their confidence in their clinical knowledge decreases, feelings of isolation and stress increase, and their preparedness to care for LGBTQIA+ patients is diminished [2, 3, 8–18].

These combined experiences illustrate how institutional culture and curriculum function as components of the “hidden curriculum,” the implicit messages students receive about which identities and patient populations are valued in medicine [20–22]. When the hidden curriculum conveys exclusion or erasure, it contributes to what the minority stress theory describes as chronic stress resulting from stigma, discrimination, and social disadvantage [23–27]. For LGBTQIA+ medical students, this stress not only undermines wellness but also diverts cognitive and emotional resources away from learning, fosters disengagement, and increases the risk of attrition [9]. Conversely, inclusive curricula and affirming learning environments can mitigate these effects, promoting psychological safety, resilience, and academic success [9].

Despite the known curricular deficiencies, reports of direct harm, and challenges with mentorship and belonging, the specific barriers and institutional factors that impact LGBTQIA+ students during their preclinical years remains unclear. Much of the existing research focuses on residents, physicians, or LGB students, leaving gaps in understanding the broader LGBTQIA+ medical student experience, particularly for transgender and gender diverse students.

This study seeks to examine how LGBTQIA+ medical students perceive their medical school’s inclusivity and interpersonal experiences, as well as how these factors affect their well-being. Findings aim to inform educators and policymakers in developing interventions that promote equity, belonging, and success within medical education.

## Methods

A 136-item, anonymous online REDCap survey was developed based on gaps in current literature regarding LGBTQIA+ medical student experiences, and informed in part by the lived experiences of authors and question reviewers. Questions were created to address the lack of comprehensive data regarding LGBTQIA+ medical student perceptions of their curriculum, interpersonal campus interactions, campus climate, and campus resources, as well as how students perceive themselves as impacted by these factors. Items were designed to capture distinct components that shape LGBTQIA+ medical student perceptions of campus inclusivity, with the goal of identifying which domains most strongly influence students’ overall sense of belonging, success, and wellbeing. Additional questions were created to explore unique aspects of inclusivity related to Osteopathic Manipulative Treatment (OMT) courses, given the absence of prior research on how LGBTQIA+ students in DO programs experience this hands-on component. The survey draft was reviewed by six LGBTQIA+ medical students of various identities and backgrounds, all of whom were external to the research team, in order to improve wording and comprehensiveness of questions.

### Survey validation

A principal components analysis (PCA) was conducted to validate the survey’s underlying structure. The Kaiser-Meyer-Olkin measure verified sampling adequacy (KMO = 0.829), and Bartlett’s test of sphericity was significant, χ²(1711) = 7133.50, *p* < .001, indicating the data’s suitability for PCA. Varimax rotation with Kaiser normalization converged in 20 iterations, producing a stable 15-component solution that explained 61.8% of the total variance. Variables with loadings of 0.40 or higher were used to interpret components. The survey demonstrated excellent internal consistency and reliability, with a Cronbach’s alpha of 0.935, supporting that the items reliably measure the intended constructs.

### Inclusion criteria

Eligible participants included students who were over 18, self-identified as LGBTQIA+, and were enrolled in accredited Doctor of Medicine (MD) or Doctor of Osteopathic Medicine (DO) institutions in the United States. To ensure adequate exposure to the curriculum and campus environment, students must have completed at least 3 months of medical school. Students in any year of medical school were eligible, although questions focused on experiences during preclinical training only.

### Survey dissemination and data collection

Students were recruited via emails sent to LGBTQIA+ student groups at medical schools across the U.S., social media posts, and through word of mouth. For schools without identifiable LGBTQIA+ student groups, recruitment emails were sent to Diversity and Inclusion or Student Affairs Offices. A total of 190 medical schools out of the 195 accredited in the U.S. were contacted by one of the means mentioned above.

Data was collected using REDcap. Informed consent was obtained, noting that participation was voluntary and confidential. No identifying information was collected, and “prefer not to say” options were provided for demographic questions to respect participants desiring further anonymity. Using SPSS Statistics for Macintosh, v.29 (IBM, Armonk, NY), a power analysis was performed based on the primary outcome of LGBTQIA+ medical students’ perceptions of campus climate and its impact on well-being. A sample size of 300 was estimated to achieve 95% power at an alpha of 0.05.

This study was approved by the institutional review boards of Philadelphia College of Osteopathic Medicine and Lewis Katz School of Medicine prior to recruitment and survey dissemination.

### Demographics

Demographic information collected included program type (DO or MD), year in school, state or territory of medical school, setting of medical school (rural, urban, or suburban), age, pronouns, gender identity, sexual orientation, and race/ethnicity. To ensure accuracy and inclusivity, participants could select all identities that applied to them, and an option to self-describe was provided for pronoun, gender identity, sexual orientation, and race/ethnicity fields.

During analysis, states were designated “red” or “blue” based upon voting trends in the last 5 presidential elections (2004–2020). Participant race/ethnicity was grouped into “underrepresented in medicine” (URIM) versus “represented in medicine” (RIM) as defined by the Association of American Medical Colleges (AAMC) [28].

Pronouns were operationalized in two ways: (1) they/them versus all other pronouns, and (2) they/them, she/they, or he/they versus all other pronouns. This approach aimed to capture differences in experiences among participants with any degree of pronoun fluidity.

Gender identity was recorded through multi-selection. A full list of identities from which participants could select from is included in Supplemental Digital Appendix 1. For analysis, participants were grouped into 4 major categories: cisgender men, cisgender women, transgender or gender-expansive (TGE), and an additional group composed of questioning/unsure individuals and those choosing not to disclose. The TGE group consisted of participants who selected agender, trans man, trans woman, transmasculine, transfeminine, genderqueer, genderfluid, nonbinary, or two-spirit, and those with queer as their only gender selection. Sexual orientation was similarly denoted through multi-selection, which can also be found in Supplemental Digital Appendix 1.

### Survey questions

Participants were asked their perception of how medical school classmates do at factors including advocacy for LGBTQIA+ inclusion, pronoun use, and inclusive terminology, using a five-point Likert scale (1 = very poorly, 5 = very well). Similar questions were asked regarding professors, with an additional question about use of inclusive lecture material. Students then ranked how well their institution addresses inclusivity, with questions on LGBTQIA+ diversity initiatives, community-building spaces, positive curriculum representation, accessible discrimination reporting, response to reports of discrimination, responsiveness to student feedback, dress code inclusivity, and access to gender-neutral bathrooms (1 = very poorly, 5 = very well). Participants also ranked each item’s impact on their overall well-being during medical school (1 = very negatively, 5 = very positively).

DO students ranked how well their Osteopathic Manipulative Treatment (OMT) department addresses several aspects of inclusivity: providing affirming dress code options, providing flexibility to choose a lab partner, emphasizing consent (enthusiastic and informed agreement), using gender-neutral terminology for body parts, and using correct pronouns (1 = very poorly, 5 = very well). Each item’s impact on well-being was then rated (1 = very negatively, 5 = very positively).

Participants then indicated whether certain adverse experiences had occurred during medical school, and with whom these events occurred (classmates, professors, or both). They also identified the presence or absence of different campus supports. Lastly, participants rated their campus climate in terms of LGBTQIA+ inclusion, and also indicated their expectations of the institution’s campus climate prior to matriculating (1 = very poor, 5 = very good). The complete survey is available as Supplemental Digital Appendix 1.

### Data analysis

Data was analyzed using SPSS Statistics for Macintosh, v.29 (IBM, Armonk, NY). We compared dependent variables across independent variables using Chi square analyses and performed paired t-tests where appropriate. A statistical significance level was set at *p* ≤ .05. For all questions, we explored differences between gender identities as well as sexual orientations to assess disparities in experiences. Given the unique challenges faced by TGE students, including potential discrimination and limited representation in medical curricula, we explored differences in experience by gender identity as well as sexual orientation. Pronoun differences were explored for questions regarding participants’ experiences with classmates and professors. Political classification and school setting were explored for questions regarding perceptions of medical school support and inclusivity. Lastly, we explored differences in MD versus DO students’ perceptions of program inclusivity and availability of campus supports, given the distinct historical and cultural approaches of these programs [29–31].

## Results

### Demographics

Six hundred twelve records were collected in REDcap, 417 of which were complete. Incomplete records were not included for analysis. 3 records were excluded due to inappropriate or intentionally misleading responses, leaving a total of 414 responses for analysis. Of the 195 incomplete records, 75 (39.8%) represented participants who opened the survey without entering data; it is unclear whether they completed the survey at a later time. Among the remaining 120 incomplete surveys, drop-offs most frequently occurred at questions regarding participant “outness” (*n* = 35, 29.2%), followed by questions on classmates, professors, and school. Among those who did not complete the survey but provided demographic information, a similar proportion identified as TGE (*n* = 22, 19.1%) compared to the proportion in our sample (*n* = 97, 23.4% TGE). Non-completers were more likely to identify as an URIM race/ethnicity (*n* = 42, 35.6%) compared to survey completers (*n* = 87, 21.0%).

Of completed surveys, 295 (71.3%) participants were in MD programs and 119 (28.7%) were in DO programs. Participants spanned all school years. 244 (58.9%) attended schools in blue states and 168 (40.6%) in red states. Five states with U.S. accredited medical schools (Mississippi-2, Montana-1, North Dakota-1, South Dakota-1, Utah-2) were not represented. Most attended schools in urban settings (*n* = 251, 60.7%), followed by suburban (*n* = 119, 28.7%) and rural (*n* = 44, 10.6%). Most participants were in their 20s (*n* = 376, 90.8%). 87 (21.0%) identified as an URIM race/ethnicity. 95 (23.0%) identified as cisgender men, 214 (51.7%) as cisgender women, and 97 (23.4%) as TGE. 84 (20.3%) participants identified as gay, 75 (18.1%) as lesbian, 197 (47.6%) as PBO, and 46 (11.1%) as queer. 2 (0.5%) participants identified as intersex and 38 (9.2%) as asexual/demisexual. Detailed demographics are in Table 1 and Supplemental Digital Appendix 2.

**Table 1:** Characteristics of 414 survey respondents from a study on LGBTQIA + U.S. medical student experiences, 2024

Characteristic	*n* (% out of 414)
Program Type	
MD	295 (71.3)
DO	119 (28.7)
Year	
M1	65 (15.7)
M2	129 (31.2)
M3	115 (27.8)
M4	105 (25.3)
State groups by historical voting pattern	
Blue	244 (58.9)
Red	168 (40.6)
Puerto Rico	1 (0.25)
Opted out	1 (0.25)
Setting	
Urban	251 (60.7)
Suburban	119 (28.7)
Rural	44 (10.6)
Age	
20s	376 (90.8)
30s+	38 (9.2)
Gender identity groups	
Cis man	95 (23.0)
Cis woman	214 (51.7)
Transgender and Gender Expansive (TGE)^a^	97 (23.4)
Additional groups (questioning, unsure, prefer not to say)	8 (1.9)
Asexual/demisexual	
Yes	38 (9.2)
No	376 (90.8)
Sexual orientation groups	
Gay	84 (20.3)
Lesbian	75 (18.1)
Pansexual, bisexual, or omnisexual (PBO)	197 (47.6)
Queer	46 (11.1)
Additional groups (Questioning, unsure, prefer not to say, asexual only)	12 (2.9)
Representation in medicine (race/ethnicity)	
Underrepresented^b^	87 (21.0)
Represented^c^	325 (78.5)
Prefer not to say	2 (0.5)
Pronouns	
He/they, She/they	57 (13.8)
They/them	30 (7.2)
She/her	223 (53.9)
He/him	103 (24.9)
Other	1 (0.25)

a. Two individuals who were in the Transgender and Gender Expansive group also identified as intersex

b. Underrepresented in medicine includes Black, Latinx, Multiethnic, American Indian/Alaska Native, Native Hawaiian/Pacific Islander, Middle Eastern or North African Native

c. Represented in medicine includes White and Asian

### Classmates

Participants indicated that classmates were worst at advocating for LGBTQIA+ inclusion, with 117 (30.0%) rating performance as poor or very poor. Classmates’ advocacy for LGBTQIA+ inclusion also had the greatest impact on participant well-being, with roughly three-quarters of participants rating it as somewhat or very impactful (*n* = 303, 77.7%). When comparing TGE to cisgender participants, statistically significant differences were found across all items, with TGE participants consistently rating classmates lower. Additionally, TGE participants rated these items as more impactful to their well-being (Table 2).

**Table 2:** 414 LGBTQIA + U.S. Medical students’ perceptions of classmate/professor inclusivity and its impact on well-being and success, 2024

Survey Item	How do your classmates do at the following?							How does it affect you?				
	Very poor *n* (%)	Poor *n* (%)	Okay *n* (%)	Well *n* (%)	Very Well *n* (%)	Cis v. TGE *p*-value ^a^	Sexual Orientation *p*-value ^b^	Neutral *n* (%)	Somewhat impactful ^c^ *n* (%)	Very impactful ^c^ *n* (%)	Cis v. TGE *p*-value ^a^	Sexual Orientation *p*-value ^b^
Inclusive terminology (*n* = 411)	7 (1.7)	62 (15.1)	148 (36.0)	138 (33.6)	56 (13.6)	**0.04**	**< 0.001**	143 (34.8)	230 (56.0)	38 (9.2)	**< 0.001**	**0.003**
Using your correct pronouns (*n* = 411)	23 (5.6)	8 (1.9)	37 (9.0)	40 (9.8)	303 (73.7)	**< 0.001**	**< 0.001**	156 (38.0)	161 (39.1)	94 (22.9)	**< 0.001**	**0.001**
Using correct pronouns for TGE individuals (*n* = 401)	29 (7.2)	84 (20.9)	139 (34.7)	111 (27.7)	38 (9.5)	**0.001**	0.09	102 (25.4)	240 (59.9)	59 (14.7)	**< 0.001**	**< 0.001**
LGBTQIA+ advocacy (*n* = 390)	24 (6.2)	93 (23.8)	117 (30.0)	102 (26.2)	54 (13.8)	**0.004**	0.054	87 (23.3)	233 (59.8)	70 (17.9)	**0.005**	**0.01**

	How do your professors do at the following?							How does it affect you?				
Survey Item	Very poor *n* (%)	Poor *n* (%)	Okay *n* (%)	Well *n* (%)	Very Well *n* (%)	Cis v. TGE *p*-value ^a^	Sexual Orientation *p*-value ^b^	Neutral *n* (%)	Somewhat impactful ^c^ *n* (%)	Very impactful ^c^ *n* (%)	Cis v. TGE *p*-value ^a^	Sexual Orientation *p*-value ^b^
Inclusive terminology (*n* = 409)	13 (3.2)	66 (16.1)	154 (37.7)	110 (26.9)	66 (16.1)	**0.04**	**< 0.001**	114 (27.9)	238 (58.2)	57 (13.9)	**0.01**	**< 0.001**
Inclusive lecture material (*n* = 413)	29 (7.0)	97 (23.5)	133 (32.2)	98 (23.7)	56 (13.6)	**0.01**	**0.02**	122 (29.5)	234 (56.7)	57 (13.8)	**< 0.001**	**0.01**
Using your correct pronouns (*n* = 407)	13 (3.2)	29 (7.1)	23 (5.7)	58 (14.2)	284 (69.8)	**< 0.001**	**< 0.001**	147 (36.1)	182 (44.7)	78 (19.2)	**< 0.001**	**< 0.001**
Using correct pronouns for TGE individuals (*n* = 375)	22 (5.9)	72 (19.2)	122 (32.5)	112 (29.9)	47 (12.5)	**< 0.001**	**0.01**	108 (28.8)	211 (56.3)	56 (14.9)	**< 0.001**	**0.01**
LGBTQIA+ advocacy (*n* = 397)	30 (7.6)	95 (23.9)	128 (32.2)	89 (22.4)	55 (13.9)	0.15	0.12	100 (25.2)	233 (58.7)	64 (16.1)	0.29	0.11

Survey items have been abbreviated. See supplemental digital appendix 1 for the complete questions. Significant *p* values appear in bold

*Abbreviations:*  *TGE* Transgender and Gender Expansive, *cis*=cisgender

a. Chi square *p*-values comparing cis v TGE. In all areas, TGE participants rated classmate and professor aspects lower than cisgender peers. TGE participants also reported greater impacts of each compared to cisgender participants

b. Chi square p-values comparing gay, lesbian, PBO, and queer. Of note, the additional sexual orientation group was excluded from analysis due to low n. Participants who identified as queer or lesbian tended to score the lowest across the various prompts Survey items have been abbreviated. See supplemental digital appendix 1 for the complete questions

c. Those who selected somewhat or very impactful came from both extremes (very poor and very well). Those who picked neutral most often came from the “okay” group with 2 exceptions, both items related to pronoun use which had the highest rating of very well amongst all items

### Professors

One hundred seventy-six (43.0%) participants indicated that professors did well or very well at using inclusive terminology regarding the LGBTQIA+ community, and 154 (37.3%) reported that professors did well or very well at providing inclusive lecture material. Statistically significant differences were found between TGE and cisgender participant ratings of inclusive terminology, inclusive lecture material, use of the students’ correct pronouns, and use of correct pronouns for TGE individuals. TGE participants rated professors as doing worse, and reported greater impact on their well-being compared to cisgender participants. Statistically significant differences were found between sexual orientation groups on questions about inclusive terminology, inclusive lecture material, use of the students’ correct pronouns, and use of correct pronouns for TGE individuals. Participants who identified as gay generally rated their professors higher compared to other sexual orientation groups, whereas those who identified as queer or lesbian reported lower ratings (Table 2).

### Medical school

One hundred fifteen (45.8%) participants rated their school as doing poorly or very poorly at taking action after reports of discrimination, microaggressions, or concerns regarding LGBTQIA+ issues, and 189 (75.3%) participants reported this impacting their well-being. Statistically significant differences were found between TGE and cisgender participant ratings of providing LGBTQIA+ specific spaces, taking action on reported discrimination, and offering affirming dress code options, with TGE participants rating their school worse across all items. Statistically significant differences emerged among sexual orientation groups regarding ratings of diversity initiatives, community-building spaces, taking action on reported discrimination, and responsiveness to student feedback. Students who identified as queer, lesbian, or PBO rated their school lower than gay participants (Table 3). 

**Table 3:** LGBTQIA+ DO/MD Students’ perceptions and impacts of medical schools and OMT departments, 2024

Survey Item	How does your school do at the following?							How does it affect you?				
	Very poor *n* (%)	Poor *n* (%)	Okay *n* (%)	Well *n* (%)	Very Well *n* (%)	Cis v. TGE *p*-value ^a^	Sexual Orientation *p*-value ^b^	Neutral *n* (%)	Somewhat impactful^c^ *n* (%)	Very impactful^c^ *n* (%)	Cis v. TGE *p*-value ^a^	Sexual Orientation *p*-value ^b^
Diversity initiatives (*n* = 410)	27 (6.6)	71 (17.3)	125 (30.5)	133 (32.4)	54 (13.2)	0.09	**< 0.001**	120 (29.3)	230 (56.1)	60 (14.6)	0.12	0.10
Providing LGBTQIA+ specific spaces (*n* = 406)	47 (11.6%)	126 (31.0)	115 (28.3)	75 (18.5)	43 (10.6)	**0.005**	**0.03**	130 (32.0%)	221 (54.5%)	55 (13.5)	**0.02**	**0.04**
Positive curricular representation (*n* = 414)	42 (10.1)	94 (22.7)	145 (35.0)	77 (18.6)	56 (13.6)	0.19	0.07	133 (32.1%)	217 (52.4%)	64 (15.5)	0.20	0.17
Reporting system for discrimination (*n* = 382)	37 (9.7)	49 (12.8)	82 (21.5)	110 (28.8)	104 (27.2)	0.11	0.67	133 (34.8)	184 (48.2)	65 (17.0)	**0.002**	**0.04**
Taking action after reported discrimination (*n* = 251)	52 (20.7)	63 (25.1)	65 (25.9)	40 (15.9)	31 (12.4)	**0.05**	**0.04**	62 (24.7)	121 (48.2)	68 (27.1)	**0.02**	**0.01**
Responsiveness to student feedback (*n* = 290)	22 (7.6)	70 (24.1)	86 (29.7)	74 (25.5)	38 (13.1)	0.19	**0.04**	91 (21.4)	150 (51.7)	49 (16.9)	0.18	**0.01**
Inclusive dress code (*n* = 265)	20 (7.5)	28 (10.6)	55 (20.8)	82 (30.9)	80 (30.2)	**0.04**	0.28	88 (33.2)	123 (46.4)	54 (20.4)	**0.04**	0.11
Inclusive bathrooms (*n* = 372)	63 (16.9)	99 (26.6)	77 (20.7)	63 (16.9)	70 (18.8)	0.35	0.14	164 (44.1)	149 (40.0)	59 (15.9)	**< 0.001**	**0.002**

DO students only = 119)	How does your OMT department do at the following?							How does it affect you?				
Survey Item	Very poor *n* (%)	Poor *n* (%)	Okay *n* (%)	Well *n* (%)	Very Well *n* (%)	Cis v. TGE *p*-value ^a^	Sexual Orientation *p*-value ^b^	Neutral *n* (%)	Somewhat impactful ^c^ *n* (%)	Very impactful ^c^ *n* (%)	Cis v. TGE *p*-value ^a^	Sexual Orientation *p*-value ^b^
Inclusive OMT lab dress code (*n* = 109)	4 (3.7)	8 (7.3)	28 (25.7)	31 (28.4)	38 (34.9)	0.23	0.75	46 (42.2)	45 (41.3)	18 (16.5)	0.09	0.90
Allowing lab partner selection (*n* = 109)	16 (14.7)	20 (18.3)	22 (20.2)	22 (20.2)	29 (26.6)	0.52	**0.04**	41 (37.6)	47 (43.1)	21 (19.3)	0.92	0.77
Emphasizing consent (*n* = 119)	3 (2.5)	9 (7.6)	12 (10.1)	21 (17.6)	74 (62.2)	0.88	0.92	17 (14.3)	55 (46.2)	47 (39.5)	0.18	0.70
Using gender-neutral terminology (*n* = 111)	9 (8.1)	17 (15.3)	33 (29.8)	32 (28.8)	20 (18.0)	0.13	**0.04**	46 (41.4)	49 (44.2)	16 (14.4)	**0.02**	0.34
Ensuring correct pronouns use (*n* = 103)	7 (6.8)	16 (15.5)	31 (30.1)	23 (22.3)	26 (25.3)	**0.003**	0.07	39 (37.9)	41 (39.8)	23 (22.3)	0.09	0.17

Survey items have been abbreviated. See supplemental digital appendix 1 for the complete questions. Significant *p* values appear in bold

*Abbreviations: OMT *Osteopathic Manipulative Treatment, *TGE *Transgender and Gender Expansive, *cis*=cisgender

a. Chi square *p*-values comparing cis v TGE. In all areas, TGE participants rated classmate and professor aspects lower than cisgender peers. TGE participants also reported greater impacts of each compared to cisgender participants

b. Chi square p-values comparing gay, lesbian, PBO, and queer. Of note, the additional sexual orientation group was excluded from analysis due to low n. Amongst medical school prompts, participants who identified as queer, lesbian, or PBO tended to score the lowest across all metrics. Amongst DO students, queer students scored their OMT departments the lowest

c. Those who selected somewhat or very impactful came from both extremes (very poor and very well). Those who picked neutral most often came from the “okay” group with 2 exceptions, both items related to pronoun use which had the highest rating of very well amongst all items

DO students reported poor LGBTQIA+ representation in their curriculum compared to MD students (*p* = .003), with this factor being more impactful to their well-being (*p* = .01). They also rated their schools more poorly at providing accessible reporting systems for discrimination (*p* < .001), with a statistically significant difference in impact on well-being *(p* < .001).

 Compared to blue states, more participants in red states rated their school as doing poorly or very poorly at providing accessible gender-neutral bathrooms (blue: *n* = 84 (38.3%), red: *n* = 78 (58.3%); *p* = .03). Similarly, more participants in rural settings rated their school as doing poorly or very poorly on this item compared to those in non-rural settings (urban: *n* = 95 (37.8%), suburban: *n* = 46 (38.6%), rural: *n* = 21 (47.7%); *p* = .04).

### OMT

Ninety five (79.8%) participants rated their OMT department as doing well or very well at emphasizing consent, with 102 (85.7%) reporting impact on well-being. Compared to cisgender students, TGE students rated their OMT departments worse at using correct pronouns for students (p = .003), and their well-being was more impacted by inadequate use of gender-neutral terminology for body parts (p = .02) (Table 3).

Students in rural settings rated their OMT departments more poorly at allowing students to choose lab partners they feel comfortable with, compared to those at urban and suburban schools (*p* = .001).

### Negative experiences

Overall, 128 (30.9%) participants reported being treated differently based on their identity by classmates (*n* = 62, 48.4%), professors (*n* = 17, 13.3%), or both (*n* = 49, 38.3%). 37 (8.9%) experienced overt harassment from classmates (*n* = 18, 48.6%), professors (*n* = 7, 18.9%), or both (*n* = 12, 32.4%). 235 (56.8%) felt that classmates (*n* = 46, 19.6%), professors (*n* = 83, 35.3%), or both (*n* = 106, 45.1%) framed LGBTQIA+ experiences as niche, exotic, or “othering.” 194 (46.9%) reported censoring their speech or mannerisms around classmates (*n* = 21, 10.8%), professors (*n* = 67, 34.5%), or both (*n* = 106, 54.6%), to avoid disclosing their identity. Students in their 30s were 2.7 times more likely to engage in self-censorship compared to students in their 20s. 319 (77.1%) participants felt compelled to educate classmates (*n* = 109, 34.2%), professors (*n* = 15, 4.7%), or both (*n* = 195, 61.1%) on LGBTQIA+ identities. 

**Fig. 1:**
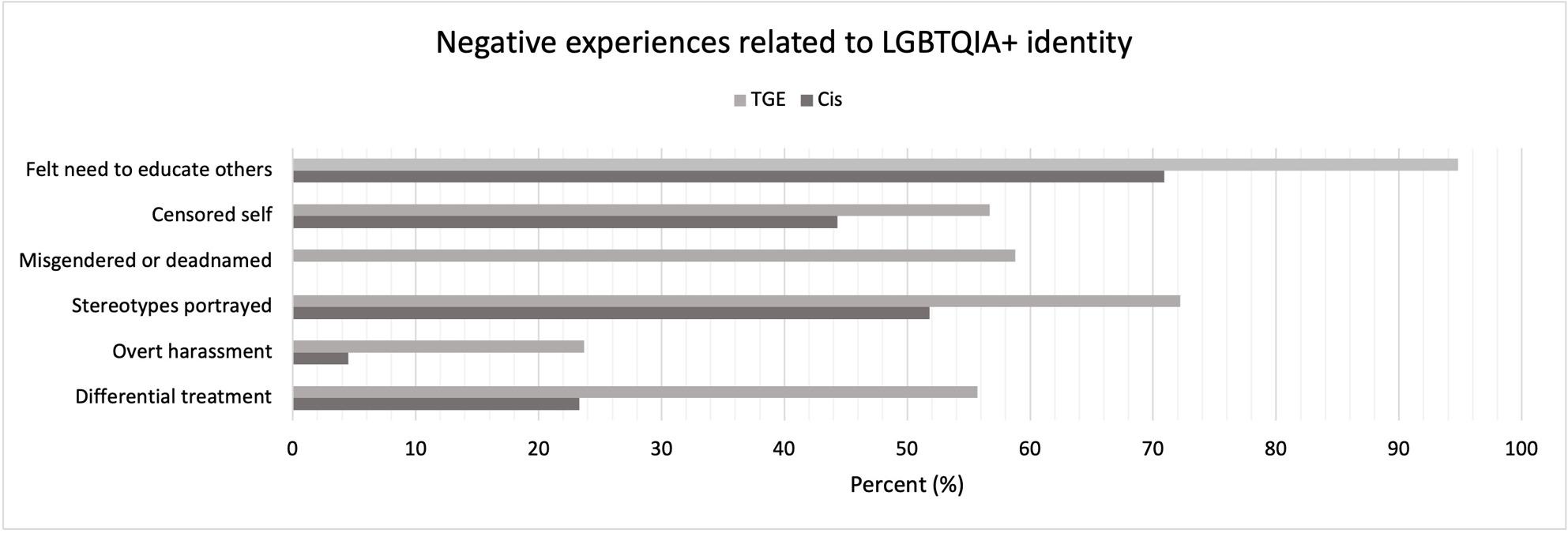
Negative Experiences of LGBTQIA+ Medical Students during Preclinical Education, 2024. Abbreviations: Transgender and Gender Expansive (TGE), cis=cisgender. The following questions were asked to cisgender (n = 309, 74.6%) and TGE (n = 97, 23.4%) participants: have you ever felt the need to educate others on your identity or other LGBTQIA+ identities, censored your speech and/or mannerisms to avoid disclosure of your LGBTQIA+ identity, felt that LGBTQIA+ experiences were presented as niche, exotic, or othering, experienced overt harassment regarding your LGBTQIA+ identity, and felt that you were treated differently due to your LGBTQIA+ identity. TGE participants were also asked if they have ever been misgendered or deadnamed

Fifty-five point seven percent (n = 54) of TGE participants reported being treated differently due to their identity and 23.7% (n = 23) experienced overt harassment. Over half (n = 57, 58.8%) reported being misgendered/deadnamed (the act of using a trans or nonbinary person’s birth name after they have changed it to reflect their gender identity), and nearly three-quarters (n = 70, 72.2%) reported that LGBTQIA+ experiences were presented as niche, exotic, or “othering.” Additionally, 56.7% (n = 55) of TGE students censored their speech or mannerisms to avoid identity disclosure, and almost all (n = 92, 94.8%) felt responsible for educating others on LGBTQIA+ identities. This data is summarized in (Fig. 1).

Similar patterns of association were found for differential treatment, overt harassment, misgendering/deadnaming, and educating others when exploring pronoun differences. Notably, 96.7% (*n* = 29) of participants who use they/them pronouns reported being misgendered/deadnamed.

Although there were no statistically significant differences between pronoun groups, participants who used he/they, she/they, or they/them pronouns were more likely to report censoring themselves or feeling that experiences are presented as niche, exotic, or othering (*p* < .1). Although not statistically significant, participants who use they/them pronouns censor themselves less than others (*n* = 11, 36.7% vs. *n* = 183, 47.6%, *p* = .25).

### Campus supports

Statistically significant differences emerged between red versus blue states and school setting, where those at schools in red states and/or rural settings were least likely to report openly-identifying LGBTQIA+ professors, inclusive terminology in the curriculum, and pride flags/symbols displayed on campus (Table 4).

**Table 4:** Presence of campus supports as indicated by 414 LGBTQIA+ U.S. medical students, 2024

Item	Yes *n* (%)	States by historical voting pattern (red v. blue) *p*-value ^a^	Setting (urban v. suburban v. rural) *p*-value ^b^
LGBTQIA+ specific club	397 (95.9)	**< 0.001**	0.51
LGBTQIA+ specific events hosted by LGBTQIA+ club	357 (86.2)	**0.002**	0.90
LGBTQIA+ specific events hosted by your institution	159 (38.4)	0.08	0.21
Other LGBTQIA+ classmates on campus	403 (97.3)	0.59	0.86
Openly supportive faculty members	373 (90.1)	0.054	0.37
Openly LGBTQIA+ faculty members	302 (72.9)	**< 0.001**	**< 0.001**
Regular use of pronouns	300 (72.5)	0.32	0.68
LGBTQIA+ inclusive terminology used in curriculum	258 (62.3)	**0.04**	**0.05**
Pride flags and symbols on campus	230 (55.6)	**0.001**	**0.05**

Significant *p* values appear in bold

a. Chi square test. Among statistically significant results, proportion is lower in red states than blue states

b. Chi square test. Among statistically significant results, proportion lowest in rural settings

 Compared to MD programs, fewer students in DO programs reported openly-identifying LGBTQIA+ faculty (MD: *n* = 232 (78.6%), DO: *n* = 70 (58.8%), *p* < .001) and inclusive terminology in their curriculum (MD: *n* = 201 (68.1%), DO: *n* = 57 (47.9%), *p* < .001).

### Campus climate

Although no statistically significant difference emerged between expectations and current rating of campus climate overall, TGE students reported significantly worse current ratings compared to expectations (prior: 3.53 (1.23), during: 3.14 (1.01); *p* = .01). TGE students also rated campus climate worse than cisgender students (cis: 3.69 (1.02), TGE: 3.14 (1.01); *p* < .001).

## Discussion

This study represents the largest survey to date examining experiences of LGBTQIA+ individuals in medical education. Its scope and depth offer a comprehensive look at how and to what extent LGBTQIA+ students are affected by the educational environment. To provide context, the 2023 AAMC Matriculation Questionnaire found that 1.4% of 14,265 matriculants into MD institutions identified as “trans man, trans woman, agender, genderqueer/non conforming, nonbinary, or another gender” and 15.9% of 14,125 medical students identify as a sexuality other than “heterosexual/straight.” [32]. Data regarding LGBTQIA+ demographics for DO matriculants is not publicly available. By addressing a wide range of experiences, interactions, and campus factors, this study captures the nuances of the realities faced by LGBTQIA+ medical students, particularly for TGE individuals.

A critical strength of this study lies in its detailed exploration of adverse experiences LGBTQIA+ medical students face, including differential treatment, overt harassment, misgendering, and self-censorship. These findings underscore systemic issues that persist despite shifting societal attitudes and institutional inclusivity efforts. The results elucidate the disproportionate burden placed on LGBTQIA+ students to educate others on their identities, which can compound feelings of marginalization and stress [33]. TGE students in particular experience significant negative impacts on their well-being during preclinical medical education.

These findings matter not only from an ethical standpoint but from an educational one. Medical schools are tasked with creating environments such that all students are supported and equipped to thrive academically and professionally. Theories such as the minority stress theory and concealable stigmatized identities suggest that chronic exposure to stigma, combined with the pressure to hide one’s identity, lead to psychological distress, cognitive fatigue, and disengagement from learning [23–27, 34]. Similarly, communities of practice theory emphasizes that learning occurs through participation and belonging; when LGBTQIA+ students feel excluded or unsafe, their participation in both formal and informal learning networks is disrupted [35]. Consequently, these experiences may hinder academic performance, reduce persistence in training, and undermining the goal of producing a competent, diverse, and empathetic physician workforce.

The study’s findings are particularly significant in the context of evolving legislation surrounding LGBTQIA+ rights. National, state, and local laws have increasingly targeted this community, restricting gender-affirming care, limiting discussions of LGBTQIA+ topics during education, and dismantling diversity, equity, and inclusion offices [36–39]. The worsening political climate will likely exacerbate the educational challenges and disparities reported in this 2024 study, heightening uncertainty and fear for LGBTQIA+ medical students, with TGE individuals being uniquely targeted and facing the greatest impact [40]. Medical institutions must recognize and respond to the impact of such legislation by establishing clear protections, offering mental health and mentorship resources, and publicly reaffirming their commitment to inclusion. If diversification of the physician workforce is a national priority, the protection and retention of LGBTQIA+ medical students must also be prioritized.

The data reveals some signs of progress. For example, 8.9% of respondents reported experiencing overt harassment by their classmates and/or professors, compared to 38% of undergraduate and graduate LGBTQIA+ students in a 2013 study [41]. In 2017, 41.7% of medical students reported anti-LGBT comments or bullying from their peers [11]. In this 2024 study, only 16.8% of respondents rated classmates “poorly” at using inclusive terminology.

However, students’ reports that their school fails to take action after discriminatory incidents makes clear that institutional accountability is often insufficient. Future research should explore what institutional responses LGBTQIA+ students view as meaningful when discrimination occurs. Qualitative, theory-driven work, perhaps grounded in restorative justice frameworks, could clarify how schools can repair trust, support learning, and restore a sense of belonging after harm.

Limitations of this study include the recruitment method; social media outreach may have disproportionately reached students who are more active in LGBTQIA+-specific spaces and emails to pride organizations could explain the high proportion of participants with campus LGBTQIA+ groups. Thus, participants may represent students with greater community support compared to LGBTQIA+ medical students overall. The proportion of white respondents (*n* = 261, 63.0%) was higher compared to AAMC’s 2023 Matriculation Survey (55.7% white) and American Association of Colleges of Osteopathic Medicine’s (AACOM) 2022 Matriculation Survey (51.8% white) [32, 42]. Additionally, five states with medical schools were not represented. Data was not collected from cisgender, heterosexual medical students, precluding comparisons to their experiences. Lastly, no correction for multiple comparisons was applied as analyses were conducted to understand these concepts for the first time.

Further studies should investigate how institutional interventions, such as inclusive curricula, responsive reporting mechanisms, and mentorship programs, affect LGBTQIA+ student well-being, engagement, and academic success over time. Qualitative and mixed-methods designs grounded in educational theory could identify mechanisms linking inclusivity with educational success, while informing actionable models for medical schools to truly support their LGBTQIA+ students.

## Conclusion

This study offers critical insights for educators, administrators, and policymakers. The results highlight a significant lack of LGBTQIA+ inclusion and institutional support for these students. The high levels of harassment and differential treatment faced by LGBTQIA+ students, and particularly TGE students, is alarming - emphasizing the urgent need for targeted interventions. Future efforts must prioritize structural changes, such as implementing more robust anti-discrimination policies and adequate reporting systems, improving LGBTQIA+ representation in curricula, and providing comprehensive support structures. Addressing these issues is urgent and essential - not just to foster equity within medical education, but also to shape a physician workforce equipped to meet the needs of diverse patient populations.

## Supplementary Information

Supplementary Material 1.

Supplementary Material 2.

## Data Availability

Publicly available data from AAMC and AACOM was used in order to compare the demographics of our participants to the broader medical student population. The datasets used and/or analysed during the current study are available from the corresponding author on reasonable request.

## Abbreviations

AACOM: American Association of Colleges of Osteopathic Medicine
AAMC: Association of American Medical Colleges
DO: Doctor of Osteopathic Medicine
LGBTQIA+: Lesbian, Gay, Bisexual, Transgender, Queer, Intersex, Asexual +
MD: Doctor of Medicine
OMT: Osteopathic Manipulative Treatment
PBO: Pansexual, bisexual, omnisexual
PCA: Principal Components Analysis
RIM: Represented in medicine
TGE: Transgender or gender-expansive
URIM: Underrepresented in medicine
