# Supplementary material for: Experiences of LGBTQIA+ medical students: gaps and challenges during preclinical education
Source: BMC Med Educ. 2026 Mar 10;26:625. doi: 10.1186/s12909-026-08966-6 (PMC13085748; doi:10.1186/s12909-026-08966-6)
Supplement: Supplementary file 1 — Supplementary Material 1. [file 12909_2026_8966_MOESM1_ESM.docx]

**Gender identity: expansive list**

| Agender, transmasculine | 1 |
| --- | --- |
| Agender, nonbinary | 1 |
| Agender, cisgender woman, queer | 1 |
| Agender, genderqueer, nonbinary | 1 |
| Agender, nonbinary, queer | 3 |
| Agender, trans woman, transfeminine, nonbinary | 2 |
| Agender, genderfluid, nonbinary, queer | 1 |
| Agender, two-spirit, queer, questioning/unsure | 1 |
| Agender, trans man, transmasculine, nonbinary, queer | 1 |
| Agender, genderqueer, genderfluid, nonbinary, intersex, queer | 1 |
| Cisgender man | 91 |
| Cisgender man, queer | 3 |
| Cisgender man, genderqueer, queer | 1 |
| Cisgender woman | 207 |
| Cisgender woman, queer | 8 |
| Cisgender woman, questioning/unsure | 3 |
| Cisgender woman, genderfluid, nonbinary | 1 |
| Cisgender woman, genderfluid, questioning/unsure | 1 |
| Cisgender woman, nonbinary, queer | 1 |
| Cisgender woman, queer, questioning/unsure | 1 |
| Trans man | 6 |
| Trans man, transmasculine | 1 |
| Trans man, queer | 1 |
| Trans man, transmasculine, nonbinary | 1 |
| Trans man, nonbinary, queer | 1 |
| Trans woman | 3 |
| Trans woman, transfeminine | 1 |
| Trans woman, not-listed/self-describe | 1 |
| Trans woman, transfeminine, nonbinary, queer | 1 |
| Transmasculine | 2 |
| Transmasculine, nonbinary | 2 |
| Transmasculine, queer | 1 |
| Transmasculine, genderqueer, nonbinary, queer | 1 |
| Transfeminine, nonbinary | 2 |
| Genderqueer | 4 |
| Genderqueer, genderfluid | 1 |
| Genderqueer, nonbinary | 1 |
| Genderqueer, queer | 1 |
| Genderqueer, questioning/unsure | 1 |
| Genderqueer, genderfluid, nonbinary | 1 |
| Genderqueer, genderfluid, queer | 2 |
| Genderqueer, queer, self-describe | 1 |
| Genderqueer, genderfluid, nonbinary, queer | 6 |
| Genderqueer, nonbinary, queer, self-describe | 1 |
| Genderfluid | 3 |
| Genderfluid, nonbinary | 1 |
| Nonbinary | 14 |
| Nonbinary, intersex | 1 |
| Nonbinary, queer | 8 |
| Two-spirit, queer | 1 |
| Queer | 7 |
| Queer, questioning/unsure | 2 |
| Questioning / unsure | 1 |
| Prefer not to answer | 1 |
| Not listed / prefer to self-describe | 2 |

**Sexual orientation: expansive list**

| Asexual | 8 |
| --- | --- |
| Asexual, bisexual | 3 |
| Asexual, gay | 1 |
| Asexual, queer | 4 |
| Asexual, questioning/unsure | 1 |
| Asexual, Not listed / prefer to self-describe | 1 |
| Asexual, bisexual, queer | 3 |
| Asexual, pansexual, queer | 1 |
| Asexual, demisexual, bisexual, queer | 2 |
| Asexual, demisexual, lesbian, queer | 1 |
| Asexual, demisexual, queer, questioning/unsure | 1 |
| Demisexual, bisexual | 3 |
| Demisexual, gay | 1 |
| Demisexual, pansexual | 1 |
| Demisexual, queer | 2 |
| Demisexual, bisexual, queer | 1 |
| Demisexual, pansexual, queer | 1 |
| Demisexual, bisexual, pansexual, queer | 1 |
| Demisexual, gay, lesbian, questioning/unsure | 1 |
| Demisexual, lesbian, queer, questioning/unsure | 1 |
| Bisexual | 80 |
| Bisexual, heterosexual/straight | 2 |
| Bisexual, lesbian | 1 |
| Bisexual, pansexual | 14 |
| Bisexual, queer | 28 |
| Bisexual, questioning/unsure | 3 |
| Bisexual, gay, queer | 2 |
| Bisexual, lesbian, queer | 1 |
| Bisexual, pansexual, queer | 14 |
| Bisexual, gay, pansexual, queer | 2 |
| Gay | 75 |
| Gay, lesbian | 2 |
| Gay, queer | 14 |
| Gay, lesbian, queer | 4 |
| Gay, pansexual, queer | 1 |
| Heterosexual / straight | 1 |
| Lesbian | 55 |
| Lesbian, pansexual | 1 |
| Lesbian, queer | 10 |
| Lesbian, pansexual, queer | 2 |
| Pansexual | 14 |
| Pansexual, queer | 14 |
| Queer | 33 |
| Questioning / unsure | 1 |
| Prefer not to answer | 1 |
| Not listed / prefer to self-describe | 1 |

**Race/ethnicity: expansive list**

| American Indian / Native American / Alaska Native | 2 |
| --- | --- |
| American Indian / Native American / Alaska Native, Black or African American | 1 |
| American Indian / Native American / Alaska Native, Hispanic / Latino/x/e | 1 |
| American Indian / Native American / Alaska Native, Middle Eastern or North African Native | 1 |
| American Indian / Native American / Alaska Native, White or Caucasian | 3 |
| American Indian / Native American / Alaska Native, Hispanic / Latino/x/e, White or Caucasian | 1 |
| American Indian / Native American / Alaska Native, Southeast Asian, White or Caucasian | 1 |
| Black or African American | 12 |
| Black or African American, White or Caucasian | 6 |
| Black or African American, Hispanic / Latino/x/e, White or Caucasian | 1 |
| East Asian | 24 |
| East Asian, Middle Eastern or North African Native | 1 |
| East Asian, Southeast Asian | 3 |
| East Asian, White or Caucasian | 7 |
| East Asian, Pacific Islander / Native Hawaiian, White or Caucasian | 1 |
| Hispanic / Latino/x/e | 17 |
| Hispanic / Latino/x/e, White or Caucasian | 19 |
| Middle Eastern or North African Native | 2 |
| Middle Eastern or North African Native, White or Caucasian | 4 |
| Pacific Islander / Native Hawaiian | 1 |
| South Asian | 32 |
| South Asian, White or Caucasian | 2 |
| Southeast Asian | 8 |
| Southeast Asian, White or Caucasian | 1 |
| White or Caucasian | 261 |
| Not listed / prefer to self-describe | 2 |
