# Supplementary material for: Experiences of LGBTQIA+ medical students: gaps and challenges during preclinical education
Source: BMC Med Educ. 2026 Mar 10;26:625. doi: 10.1186/s12909-026-08966-6 (PMC13085748; doi:10.1186/s12909-026-08966-6)
Supplement: Supplementary file 2 — Supplementary Material 2. [file 12909_2026_8966_MOESM2_ESM.pdf]

Understanding Experiences of LGBTQIA+ Medical Students During Didactic Undergraduate Medical Education

Thank you for your interest in this research survey!

We are a team of queer and trans medical students from the Philadelphia College of Osteopathic Medicine and the Lewis Katz School of Medicine at Temple University. We are asking for your consent to take part in this study. This page describes the key information that we believe most people need to decide whether to consent to taking part in this research.

The goal of this study is to understand unique experiences that affect the well being and success of LGBTQIA+ medical students across United States medical schools during their didactic years (preclinical years spent in the classroom). Our findings will provide valuable information that can help direct LGBTQIA+ programming and inclusion initiatives at medical schools across the country.

To participate, you must:

Identify as LGBTQIA+Be enrolled in a US medical school (MD or DO)Have completed at least 3 months of medical schoolBe willing and able to complete a 10-minute surveyThis survey will ask about your experiences with various aspects of medical school campus climate, curriculum, and interactions with classmates and faculty. Please be aware that some of the questions asked may pertain to experiences that are emotionally sensitive or triggering. Participation is voluntary and you may exit the survey at any time. This survey contains multiple choice and Likert-scale questions with a few opportunities to expand if needed.

Confidentiality:

Your participation in this research will be held strictly confidential to the extent possible. However, confidentiality cannot be guaranteed, and therefore there is a mild risk of breach of confidentiality. We will be collecting basic demographic information, but your name will not be associated with your responses. Your responses may be shared with individuals and organizations that conduct or oversee this research, including:

Research personnel at both the Philadelphia College of Medicine and the Lewis Katz School of Medicine at Temple UniversityThe Institutional Review Board (IRB) that reviewed this researchTemple UniversityPhiladelphia College of Osteopathic MedicineTemple University Health System and its affiliatesWe may publish the results of this research. However, we will keep any identifying information confidential. We may de-identify this data and share it with other researchers for research that is currently unknown. All data will be destroyed 3 years after completion of the study per institutional policies.

Questions?

If you have questions, concerns, or feedback regarding the survey, please contact us at LGBTQIAMedStudentPrj@gmail.com or (215) 707-3559.

This research is being overseen by an Institutional Review Board ("IRB"). An IRB is a group of people who perform independent review of research studies. You may talk to them at (215) 707-3390 or irb@temple.edu if you have questions, concerns, or complaints that are not being answered by the research team, or if you have questions about your rights as a research subject.

Temple Protocol No.: 31513

PCOM Protocol No.: H24-033X

Principal Investigators: LeeAnn Tanaka, DO and Robert Bettiker, MD

	◯ I consent to participate<br>◯ I do not consent to participate
MD/DO Program	◯ MD<br>◯ DO

Year	◯ MS 1<br>◯ MS 2<br>◯ MS 3<br>◯ MS 4
M1 students are eligible if they have been in medical school for at least 3 months. Have you been in medical school for at least 3 months?	◯ Yes ◯ No

State/territory of medical school	◯ Prefer not to answer<br>◯ Alabama<br>◯ Alaska<br>◯ Arizona<br>◯ Arkansas<br>◯ California<br>◯ Colorado<br>◯ Connecticut<br>◯ District of Columbia<br>◯ Delaware<br>◯ Florida<br>◯ Georgia<br>◯ Hawaii<br>◯ Idaho<br>◯ Illinois<br>◯ Indiana<br>◯ Iowa<br>◯ Kansas<br>◯ Kentucky<br>◯ Louisiana<br>◯ Maine<br>◯ Maryland<br>◯ Massachusetts<br>◯ Michigan<br>◯ Minnesota<br>◯ Mississippi<br>◯ Missouri<br>◯ Montana<br>◯ Nebraska<br>◯ Nevada<br>◯ New Hampshire<br>◯ New Jersey<br>◯ New Mexico<br>◯ New York<br>◯ North Carolina<br>◯ North Dakota<br>◯ Ohio<br>◯ Oklahoma<br>◯ Oregon<br>◯ Pennsylvania<br>◯ Puerto Rico<br>◯ Rhode Island<br>◯ South Carolina<br>◯ South Dakota<br>◯ Tennessee<br>◯ Texas<br>◯ Utah<br>◯ Vermont<br>◯ Virginia<br>◯ Washington<br>◯ West Virginia<br>◯ Wisconsin<br>◯ Wyoming
Setting of medical school	◯ Urban<br>◯ Suburban<br>◯ Rural
Age	◯ 18-20<br>◯ 21-24<br>◯ 25-29<br>◯ 30-34<br>◯ 35+

Pronouns

____

Gender Identity
Check all that apply

- ☐ Agender
- ☐ Cisgender man
- ☐ Cisgender woman
- ☐ Trans man
- ☐ Trans woman
- ☐ Transmasculine
- ☐ Transfeminine
- ☐ Genderqueer
- ☐ Genderfluid
- ☐ Nonbinary
- ☐ Intersex
- ☐ Two-spirit
- ☐ Queer
- ☐ Questioning / unsure
- ☐ Prefer not to answer
- ☐ Not listed / prefer to self-describe

If you would like to self-describe, please do so here:

____

Sexual Orientation
Check all that apply

- ☐ Asexual
- ☐ Demisexual
- ☐ Bisexual
- ☐ Gay
- ☐ Heterosexual / straight
- ☐ Lesbian
- ☐ Pansexual
- ☐ Queer
- ☐ Questioning / unsure
- ☐ Prefer not to answer
- ☐ Not listed / prefer to self-describe

If you would like to self-describe, please do so here:

____

Race
Check all that apply

- ☐ American Indian / Native American / Alaska Native
- ☐ Black or African American
- ☐ East Asian
- ☐ Hispanic / Latino/x/e
- ☐ Middle Eastern or North African Native
- ☐ Pacific Islander / Native Hawaiian
- ☐ South Asian
- ☐ Southeast Asian
- ☐ White or Caucasian
- ☐ Prefer not to answer
- ☐ Not listed / prefer to self-describe

If you would like to self-describe, please do so here:

____

Before starting medical school, what proportion of people in these groups do you think were aware of your LGBTQIA+ identity?

	None	Some	Most	All
Members of your immediate family (e.g., parents, siblings)	○	○	○	○
Members of your extended family (e.g., aunts, uncles, grandparents, cousins)	○	○	○	○
People you socialize with (e.g., friends)	○	○	○	○
People at work/school (e.g., coworkers, supervisors, instructors, students)	○	○	○	○
Strangers (e.g., someone you have a casual conversation with that you had not previously met)	○	○	○	○

Before starting medical school, how often did you avoid talking about topics related to or otherwise indicating your LGBTQIA+ identity when interacting with members of these groups? (e.g., not talking about your significant other, changing your mannerisms, etc.)

	Never	Some of the time	Most of the time	All the time
Members of your immediate family (e.g., parents, siblings)	◯	◯	◯	◯
Members of your extended family (e.g., aunts, uncles, grandparents, cousins)	◯	◯	◯	◯
People you socialize with (e.g., friends)	◯	◯	◯	◯
People at work/school (e.g., coworkers, supervisors, instructors, students)	◯	◯	◯	◯
Strangers (e.g., someone you have a casual conversation with that you had not previously met)	◯	◯	◯	◯

The following questions pertain to your support system:

	Never	Some of the time	Most of the time	All of the time
Before starting medical school, how often did you have a support system to lean on during difficult times?	○	○	○	○
While at medical school, how often do you have a support system to lean on during difficult times?	○	○	○	○

* Writing in blue correlates with the terminology used in table 2

During preclinical years of medical school, how do you think your classmates do/did at the following:

	Very Poorly	Poorly	Okay	Well	Very Well	Unsure
Using inclusive terminology regarding the LGBTQIA+ community, patients, or experiences (Inclusive terminology)	◯	◯	◯	◯	◯	◯

	Very negatively	Negatively	Neutral	Positively	Very positively
In your opinion, how does the above item affect your overall well being and success during medical school?	◯	◯	◯	◯	◯

	Very Poorly	Poorly	Okay	Well	Very Well	Unsure
Using the correct pronouns to refer to you (Using your correct pronouns)	◯	◯	◯	◯	◯	◯

	Very negatively	Negatively	Neutral	Positively	Very positively
In your opinion, how does the above item affect your overall well being and success during medical school?	◯	◯	◯	◯	◯

	Very Poorly	Poorly	Okay	Well	Very Well	Unsure
Using the correct pronouns to refer to trans, nonbinary, genderfluid individuals or patients (Using correct pronouns for TGE individuals)	◯	◯	◯	◯	◯	◯

	Very negatively	Negatively	Neutral	Positively	Very positively
In your opinion, how does the above item affect your overall well being and success during medical school?	◯	◯	◯	◯	◯

	Very Poorly	Poorly	Okay	Well	Very Well	Unsure
Advocating for LGBTQIA+ inclusion (e.g., speaking up in class to protect LGBTQIA+ students, correcting a faculty member when they misgender a student) (LGBTQIA+ Advocacy)	◯	◯	◯	◯	◯	◯

	Very negatively	Negatively	Neutral	Positively	Very positively
In your opinion, how does the above item affect your overall well being and success during medical school?	◯	◯	◯	◯	◯

*** Writing in blue correlates with the terminology used in table 2**

During preclinical years of medical school, how do you think your professors and faculty members do/did at the following:

	Very Poorly	Poorly	Okay	Well	Very Well	Unsure
Using inclusive terminology regarding the LGBTQIA+ community, patients, or experiences (Inclusive terminology)	○	○	○	○	○	○

	Very negatively	Negatively	Neutral	Positively	Very positively
In your opinion, how does the above item affect your overall well being and success during medical school?	○	○	○	○	○

	Very Poorly	Poorly	Okay	Well	Very Well	Unsure
Providing lecture material with inclusive terminology regarding the LGBTQIA+ community, patients, or experiences (Inclusive lecture material)	○	○	○	○	○	○

	Very negatively	Negatively	Neutral	Positively	Very positively
In your opinion, how does the above item affect your overall well being and success during medical school?	○	○	○	○	○

	Very Poorly	Poorly	Okay	Well	Very Well	Unsure
Using the correct pronouns to refer to you (Using your correct pronouns)	○	○	○	○	○	○

	Very negatively	Negatively	Neutral	Positively	Very positively
In your opinion, how does the above item affect your overall well being and success during medical school?	○	○	○	○	○

	Very Poorly	Poorly	Okay	Well	Very Well	Unsure
Using the correct pronouns to refer to trans, nonbinary, genderfluid individuals or patients (Using correct pronouns for TGE individuals)	○	○	○	○	○	○

	Very negatively	Negatively	Neutral	Positively	Very positively
In your opinion, how does the above item affect your overall well being and success during medical school?	○	○	○	○	○

	Very Poorly	Poorly	Okay	Well	Very Well	Unsure

Advocating for LGBTQIA+ inclusion (e.g., correcting student assumptions about the LGBTQIA+ community, working to increase overall curricular inclusivity)<br>(LGBTQIA+ advocacy)	◯	◯	◯	◯	◯	◯

	Very negatively	Negatively	Neutral	Positively	Very positively
In your opinion, how does the above item affect your overall well being and success during medical school?	◯	◯	◯	◯	◯

* Writing in blue correlates with the terminology used in table 3

How do you think your medical school does at the following:

	Very Poorly	Poorly	Okay	Well	Very Well	Unsure
Creating diversity and inclusion initiatives that support LGBTQIA+ students (Diversity initiatives)	○	○	○	○	○	○

	Very negatively	Negatively	Neutral	Positively	Very positively
In your opinion, how does the above item affect your overall well being and success during medical school?	○	○	○	○	○

	Very Poorly	Poorly	Okay	Well	Very Well	Unsure
Providing LGBTQIA+ specific spaces for students and faculty to build community (Providing LGBTQIA+ specific spaces)	○	○	○	○	○	○

	Very negatively	Negatively	Neutral	Positively	Very positively
In your opinion, how does the above item affect your overall well being and success during medical school?	○	○	○	○	○

	Very Poorly	Poorly	Okay	Well	Very Well	Unsure
Incorporating positive LGBTQIA+ representation within the curriculum (including SP cases, lecture material, etc.) (Positive curricular representation)	○	○	○	○	○	○

	Very negatively	Negatively	Neutral	Positively	Very positively
In your opinion, how does the above item affect your overall well being and success during medical school?	○	○	○	○	○

	Very Poorly	Poorly	Okay	Well	Very Well	Unsure
Providing an accessible reporting system for overt discrimination or microaggressions (Reporting system for discrimination)	○	○	○	○	○	○

	Very negatively	Negatively	Neutral	Positively	Very positively
In your opinion, how does the above item affect your overall well being and success during medical school?	○	○	○	○	○

	Very Poorly	Poorly	Okay	Well	Very Well	Unsure
Taking action after discrimination, microaggressions, or concerns regarding LGBTQIA+ issues are reported (Taking action after reported discrimination)	○	○	○	○	○	○

	Very negatively	Negatively	Neutral	Positively	Very positively
In your opinion, how does the above item affect your overall well being and success during medical school?	◯	◯	◯	◯	◯

	Very Poorly	Poorly	Okay	Well	Very Well	Unsure
Responsiveness to student feedback regarding LGBTQIA+ inclusivity on campus (Responsiveness to student feedback)	◯	◯	◯	◯	◯	◯

	Very negatively	Negatively	Neutral	Positively	Very positively
In your opinion, how does the above item affect your overall well being and success during medical school?	◯	◯	◯	◯	◯

	Very Poorly	Poorly	Okay	Well	Very Well	Unsure
Providing dress code options that align with your LGBTQIA+ identity and expression (Inclusive dress code)	◯	◯	◯	◯	◯	◯

	Very negatively	Negatively	Neutral	Positively	Very positively
In your opinion, how does the above item affect your overall well being and success during medical school?	◯	◯	◯	◯	◯

	Very Poorly	Poorly	Okay	Well	Very Well	Unsure
Providing gender-neutral bathrooms on campus that are accessible at all times (Inclusive bathrooms)	◯	◯	◯	◯	◯	◯

	Very negatively	Negatively	Neutral	Positively	Very positively
In your opinion, how does the above item affect your overall well being and success during medical school?	◯	◯	◯	◯	◯

*** Writing in blue correlates with the terminology used in table 3**

In terms of OMT lab, how do you think your OMT department does/did at the following:

	Very Poorly	Poorly	Okay	Well	Very Well	N/A
Providing OMT lab dress code options that allow you to present in alignment with your gender (Inclusive OMT lab dress code)	◯	◯	◯	◯	◯	◯

	Very negatively	Negatively	Neutral	Positively	Very positively
In your opinion, how does the above item affect your overall well being and success during medical school?	◯	◯	◯	◯	◯

	Very Poorly	Poorly	Okay	Well	Very Well	N/A
Allowing you to select a lab partner that you feel comfortable working with (Allowing lab partner selection)	◯	◯	◯	◯	◯	◯

	Very negatively	Negatively	Neutral	Positively	Very positively
In your opinion, how does the above item affect your overall well being and success during medical school?	◯	◯	◯	◯	◯

	Very Poorly	Poorly	Okay	Well	Very Well	N/A
Emphasizing consent	◯	◯	◯	◯	◯	◯

	Very negatively	Negatively	Neutral	Positively	Very positively
In your opinion, how does the above item affect your overall well being and success during medical school?	◯	◯	◯	◯	◯

	Very Poorly	Poorly	Okay	Well	Very Well	N/A
Using gender-neutral terminology for body parts (Using gender-neutral terminology)	◯	◯	◯	◯	◯	◯

	Very negatively	Negatively	Neutral	Positively	Very positively
In your opinion, how does the above item affect your overall well being and success during medical school?	◯	◯	◯	◯	◯

	Very Poorly	Poorly	Okay	Well	Very Well	N/A
Ensuring correct pronouns are used for students	◯	◯	◯	◯	◯	◯

	Very negatively	Negatively	Neutral	Positively	Very positively
In your opinion, how does the above item affect your overall well being and success during medical school?	◯	◯	◯	◯	◯

At your medical school, how comfortable are you:

	Not at all	Somewhat	Very	N/A
Being "out" as LGBTQIA+ amongst your classmates?	○	○	○	○
Being "out" as LGBTQIA+ amongst professors/faculty? (e.g., through clubs, events, conversation, etc.)	○	○	○	○
Using your correct pronouns with classmates?	○	○	○	○
Using your correct pronouns with professors/faculty?	○	○	○	○
Presenting in alignment with your identity on campus (when NOT required to follow a dress code)?	○	○	○	○
Presenting in alignment with your identity when required to follow a specific dress code?	○	○	○	○

During preclinical years of medical school, have you ever:	
Felt that you were treated differently due to your LGBTQIA+ identity?	◯ Yes ◯ No
By a	☐ Classmate ☐ Professor/faculty member
Experienced overt harassment regarding your LGBTQIA+ identity?	◯ Yes ◯ No
By a	☐ Classmate ☐ Professor/faculty member
Felt that LGBTQIA+ experiences were presented as niche, exotic, or othering?	◯ Yes ◯ No
By a	☐ Classmate ☐ Professor/faculty member
Been misgendered or deadnamed?	◯ Yes ◯ No ◯ N/A
By a	☐ Classmate ☐ Professor/faculty member
Censored your speech and/or mannerisms to avoid disclosure of your LGBTQIA+ identity?	◯ Yes ◯ No
Around	☐ Classmates ☐ Professor/faculty member
Felt the need to educate others on your identity or other LGBTQIA+ identities?	◯ Yes ◯ No
Around	☐ Classmates ☐ Professor/faculty member

The following questions pertain to your overall experience:

	Very poor	Poor	Neutral	Good	Very good	Unsure
Prior to starting medical school, how did you expect the campus climate of your medical school to be in terms of LGBTQIA+ inclusion?	○	○	○	○	○	○
As a current medical student, how would you rate the campus climate in terms of LGBTQIA+ inclusion?	○	○	○	○	○	○

Which of the following aspects, if any, do you think have been negatively affected due to your experiences as an LGBTQIA+ student on campus?

Check all that apply

- ☐ Overall academic success
- ☐ Overall mental health
- ☐ Class attendance
- ☐ Engagement in class
- ☐ Sense of connection to classmates
- ☐ Sense of belonging at your school
- ☐ Resilience after expected medical school setbacks
- ☐ Participation in extracurricular activities at school
- ☐ Ability to network with MD/DO mentors
- ☐ Confidence in my LGBTQIA+ identity overall
- ☐ Confidence in disclosing my LGBTQIA+ identity on residency applications
- ☐ Confidence that my classmates are prepared to care for LGBTQIA+ patients
- ☐ Confidence in pursuing the specialty that aligns with my interests
- ☐ Confidence in continuing to pursue a career in medicine
- ☐ None of these have been negatively affected

If there are additional areas that you believe were negatively affected due to your experiences as an LGBTQIA+ student that were are not listed above, please share here:

____

At any point during preclinical years of medical school, did you ever consider transferring or dropping out as a result of your experiences as a LGBTQIA+ identifying student?

- ○ Yes
- ○ No

Please indicate whether the following exist on your campus:

LGBTQIA+ specific club	◯ Yes ◯ No ◯ Unsure
How important has this been in supporting your wellbeing and success as a LGBTQIA+ medical student?	◯ Not at all important<br>◯ Somewhat important<br>◯ Very important
If this did exist on your campus, how important do you think it would be in supporting your wellbeing and success as a LGBTQIA+ medical student?	◯ Not at all important<br>◯ Somewhat important<br>◯ Very important
LGBTQIA+ specific events hosted by your campus LGBTQIA+ club	◯ Yes ◯ No ◯ Unsure
How important has this been in supporting your wellbeing and success as a LGBTQIA+ medical student?	◯ Not at all important<br>◯ Somewhat important<br>◯ Very important
If this did exist on your campus, how important do you think it would be in supporting your wellbeing and success as a LGBTQIA+ medical student?	◯ Not at all important<br>◯ Somewhat important<br>◯ Very important
LGBTQIA+ specific events hosted by your institution	◯ Yes ◯ No ◯ Unsure
How important has this been in supporting your wellbeing and success as a LGBTQIA+ medical student?	◯ Not at all important<br>◯ Somewhat important<br>◯ Very important
If this did exist on your campus, how important do you think it would be in supporting your wellbeing and success as a LGBTQIA+ medical student?	◯ Not at all important<br>◯ Somewhat important<br>◯ Very important
Other LGBTQIA+ classmates on campus	◯ Yes ◯ No ◯ Unsure
How important has this been in supporting your wellbeing and success as a LGBTQIA+ medical student?	◯ Not at all important<br>◯ Somewhat important<br>◯ Very important
If this did exist on your campus, how important do you think it would be in supporting your wellbeing and success as a LGBTQIA+ medical student?	◯ Not at all important<br>◯ Somewhat important<br>◯ Very important
Staff/faculty members that are openly supportive of the LGBTQIA+ community	◯ Yes ◯ No ◯ Unsure
How important has this been in supporting your wellbeing and success as a LGBTQIA+ medical student?	◯ Not at all important<br>◯ Somewhat important<br>◯ Very important
If this did exist on your campus, how important do you think it would be in supporting your wellbeing and success as a LGBTQIA+ medical student?	◯ Not at all important<br>◯ Somewhat important<br>◯ Very important
Openly LGBTQIA+ identifying staff/faculty members	◯ Yes ◯ No ◯ Unsure

How important has this been in supporting your wellbeing and success as a LGBTQIA+ medical student?	◯ Not at all important<br>◯ Somewhat important<br>◯ Very important
If this did exist on your campus, how important do you think it would be in supporting your wellbeing and success as a LGBTQIA+ medical student?	◯ Not at all important<br>◯ Somewhat important<br>◯ Very important
Regular use of pronouns (on badges, Zoom calls, email signatures, etc.)	◯ Yes ◯ No ◯ Unsure
How important has this been in supporting your wellbeing and success as a LGBTQIA+ medical student?	◯ Not at all important<br>◯ Somewhat important<br>◯ Very important
If this did exist on your campus, how important do you think it would be in supporting your wellbeing and success as a LGBTQIA+ medical student?	◯ Not at all important<br>◯ Somewhat important<br>◯ Very important
Adequate education about LGBTQIA+ health and patient care in the curriculum	◯ Yes ◯ No ◯ Unsure
How important has this been in supporting your wellbeing and success as a LGBTQIA+ medical student?	◯ Not at all important<br>◯ Somewhat important<br>◯ Very important
If this did exist on your campus, how important do you think it would be in supporting your wellbeing and success as a LGBTQIA+ medical student?	◯ Not at all important<br>◯ Somewhat important<br>◯ Very important
LGBTQIA+ inclusive terminology used in curriculum	◯ Yes ◯ No ◯ Unsure
How important has this been in supporting your wellbeing and success as a LGBTQIA+ medical student?	◯ Not at all important<br>◯ Somewhat important<br>◯ Very important
If this did exist on your campus, how important do you think it would be in supporting your wellbeing and success as a LGBTQIA+ medical student?	◯ Not at all important<br>◯ Somewhat important<br>◯ Very important
Access to LGBTQIA+ inclusive counseling / mental health services	◯ Yes ◯ No ◯ Unsure
How important has this been in supporting your wellbeing and success as a LGBTQIA+ medical student?	◯ Not at all important<br>◯ Somewhat important<br>◯ Very important
If this did exist on your campus, how important do you think it would be in supporting your wellbeing and success as a LGBTQIA+ medical student?	◯ Not at all important<br>◯ Somewhat important<br>◯ Very important
Pride flags and symbols displayed on campus	◯ Yes ◯ No ◯ Unsure
How important has this been in supporting your wellbeing and success as a LGBTQIA+ medical student?	◯ Not at all important<br>◯ Somewhat important<br>◯ Very important

If this did exist on your campus, how important do you think it would be in supporting your wellbeing and success as a LGBTQIA+ medical student?	◯ Not at all important<br>◯ Somewhat important<br>◯ Very important
Adequate peer mentorship program	◯ Yes ◯ No ◯ Unsure
How important has this been in supporting your wellbeing and success as a LGBTQIA+ medical student?	◯ Not at all important<br>◯ Somewhat important<br>◯ Very important
If this did exist on your campus, how important do you think it would be in supporting your wellbeing and success as a LGBTQIA+ medical student?	◯ Not at all important<br>◯ Somewhat important<br>◯ Very important
Adequate faculty mentorship program	◯ Yes ◯ No ◯ Unsure
How important has this been in supporting your wellbeing and success as a LGBTQIA+ medical student?	◯ Not at all important<br>◯ Somewhat important<br>◯ Very important
If this did exist on your campus, how important do you think it would be in supporting your wellbeing and success as a LGBTQIA+ medical student?	◯ Not at all important<br>◯ Somewhat important<br>◯ Very important
If there are other things that have felt supportive for you regarding your LGBTQIA+ identity during your pre-clerkship years, please share here:	______________________________
If there is anything else you would like to add, please do so here:	______________________________
